# A computational framework for identifying chemical compounds to bind Apolipoprotein E4 for Alzheimer’s disease intervention

**DOI:** 10.3389/fsysb.2023.1188430

**Published:** 2023-06-14

**Authors:** Tianhua Zhai, Emily Krass, Fangyuan Zhang, Zuyi Huang

**Affiliations:** Department of Chemical and Biological Engineering, Villanova University, Villanova, PA, United States

**Keywords:** Alzherimer’s disease, protein interacting network, systems biolgoy, computational drug discovery, drug target, chemical compound

## Abstract

Alzheimer’s disease (AD), a neurodegenerative disorder, is characterized by its ability to cause memory loss and damage other cognitive functions. Aggregation of amyloid beta (Aβ) plaques and neurofibrillary tangles in the brain are responsible for the development of Alzheimer’s disease (AD). While attempts targeting Aβ and tau proteins have been extensively conducted in the past decades, only two FDA-approved drugs (i.e., monoclonal antibodies) tackle the underlying biology of Alzheimer’s disease. In this study, an integrated computational framework was developed to identify new drug targets for Alzheimer’s disease and identify small molecules as potential therapeutical options. A systematic investigation of the gene networks firstly revealed that the Apolipoprotein E4 (ApoE4) gene plays a central role among genes associated with Alzheimer’s disease. The ApoE4 protein was then chosen as the protein target based on its role in the main pathological hallmarks of AD, which has been shown to increase Aβ accumulation by directly binding to Aβ as well as interfering with Aβ clearance that is associated with other receptors. A library of roughly 1.5 million compounds was then virtually screened via a ligand-protein docking program to identify small-molecule compounds with potential binding capacity to the ApoE4 N-terminal domain. On the basis of compound properties, 312 compounds were selected, analyzed and clustered to further identify common structures and essential functional groups that play an important role in binding ApoE4. The *in silico* prediction suggested that compounds with four common structures of sulfon-amine-benzene, 1,2-benzisothiazol-3-amine 1,1-dioxide, N-phenylbenzamide, and furan-amino-benzene presented strong hydrogen bonds with residues E27, W34, R38, D53, D153, or Q156 in the N terminal of ApoE4. These structures might also form strong hydrophobic interactions with residues W26, E27, L28, L30, G31, L149, and A152. While the 312 compounds can serve as drug candidates for further experiment assays, the four common structures, along with the residues for hydrogen bond or hydrophobic interaction, pave the foundation to further optimize the compounds as better binders of ApoE4.

## 1 Introduction

On 3 November 1906, the first case of Alzheimer’s disease (AD) was reported by Alois Alzheimer, a clinical psychiatrist and neuroanatomist at Munich University Hospital (Hippius and Neundörfer, 2003). Since people can live longer than in the past, Alzheimer’s disease has become a major public health issue in the world. According to “2022 Alzheimer’s Disease Facts and Figures” reported by Alzheimer’s Association, an estimated 6.5 million Americans are living with Alzheimer’s dementia in 2022, and the number of AD Americans is projected to double in 2050 (Alzheimer’s Association, 2022). Alzheimer’s disease was officially listed as the sixth-leading cause of death in the United States in 2019. The U.S. Annual Alzheimer’s death rate was generally increasing by year from 2000 to 2019 (Alzheimer’s Association, 2022). Alzheimer’s disease has become the most common cause of dementia, with the most symptoms of memory loss that disrupt daily life, challenges in planning or solving problems, and difficulty in completing familiar tasks (Markus, 2020), (Alzheimer’s Association, 2020). All these indicate an urgent need to investigate effective interventions for Alzheimer’s disease.

Extensive research has been conducted on Alzheimer’s disease in the last few decades. It is reported that the pathology of Alzheimer’s disease is characterized by the formation of amyloid plaques and tau protein tangles in patients’ brains (LaFerla and Oddo, 2005; Li, 2018; Wu, 2021). Amyloid plaques accumulated between neuron cells interrupt the communication of these cells (Huffels, 2022). Tau proteins are the most frequent microtubule-associated proteins in the neurons of the central nervous system (CNS). The tau tangles affect the maintenance of metabolism of neuron cells. The accumulation of amyloid plaques and tau protein tangles, which eventually causes the death of neuron cells, is a slow process. It may take 20 years or longer before the patients show AD symptoms that interfere with some everyday activities. The early-onset patients, who generally have a family history of Alzheimer’s disease, show symptoms before the age of 65. These familial patients account for only 5% of AD cases. The other patients who have no family history of Alzheimer’s disease (so-called sporadic patients) generally show AD symptoms after age 65. After the initial diagnosis of Alzheimer’s disease, most people live with it for between 3 and 11 years.

Since the progression of Alzheimer’s disease is slow, it is important to identify biomarkers that can be used as potential indicators for early diagnosis. It is reported that familial patients generally carry genes APP (amyloid precursor protein), PSEN1 (Presenilin-1), and PSEN2 (Presenilin-1) (Tanzi, 2012; Lanoiselée, 2017; Gao, 2019). All these genes are involved in the synthesis of amyloid. While extensive studies have been conducted to identify biomarker genes for sporadic patients, who account for 95% of AD cases (Grünblatt, 2009; Lista, 2015; Castanho and Lunnon, 2019), no common biomarker genes like APP, PSEN1, and PSEN2 for familial patients have been identified. This explains the challenges in drug discovery to treat AD patients. Only seven drugs have been approved by the FDA to treat Alzheimer’s disease, including Lecanemab (Swanson, 2021), Aducanumab (Schneider, 2020), Donepezil (Birks et al., 2000), Rivastigmine (Desai and Grossberg, 2005), Memantine (Reisberg et al., 2003), Manufactured combination of memantine and donepezil (Howard, 2012), and Galantamine (Lilienfeld, 2002). Five of these seven drugs are designed to reduce AD symptoms (e.g., memory loss and confusion), and enable AD patients to maintain certain daily functions (Birks et al., 2000; Lilienfeld, 2002; Reisberg et al., 2003; Desai and Grossberg, 2005; Howard, 2012). They are unable to stop the disease from worsening over time. Among these seven drugs, only Lecanemab and Aducanumab, monoclonal antibodies, tackle the underlying biology of Alzheimer’s disease by reducing the accumulation of amyloid plaques (Sevigny, 2016). Both of them were approved by the FDA for Biogen recently under the FDA’s Accelerated Approval pathway. However, Aducanumab was studied in people living with early Alzheimer’s disease, without safety or effectiveness data on initiating treatment at earlier or later stages of the disease than were studied (Dhillon, 2021), (Budd Haeberlein, 2022). The accelerated approval of accumulation is controversial (Vaz et al., 2022). Concerns were raised for the side effects of Lecanemab. New drugs to treat Alzheimer’s disease are still in high demand.

To address the aforementioned knowledge gap, a pipeline integrating systems biology and computational drug discovery was developed in this work. The pipeline is built upon existing computational tools and databases to accelerate the pace of drug discovery for Alzheimer’s disease. In particular, DisGeNet (Piñero, 2017), STRING (Szklarczyk, 2021), Cytoscape (Shannon, 2003), MCODE (Bader and Hogue, 2003), cytoHubba (Chin et al., 2014), DAVID (Dennis, 2003), all of which are free commonly-used tools in systems biology, were integrated to identify genes associated with Alzheimer’s disease in medical databases (via DisGeNet), generate interaction networks of proteins encoded by the selected genes (via STRING), visualize the protein-protein interaction networks (via Cytoscape), investigate the highly-interactive subnetworks of proteins (via MCODE), rank the protein targets in subnetworks based on their interactions with other proteins (via Cytohubba), and further study the biological meanings behind the top-ranked protein targets (via DAVID). DisGeNET is one of the largest public databases of human disease-related genes and variations (Piñero, 2017). STRING is a database of known and predicted protein-protein interactions that covers 24 million proteins from 5,090 organisms (Szklarczyk, 2021). Cytoscape was chosen not only because it is a common platform used for visualizing complex networks but also it integrates those networks with programs for subnetwork analysis and protein target ranking (Shannon, 2003).

The top protein targets selected through our pipeline were further screened based on whether crystal structures are available. One of these targets is Apolipoprotein E4 (ApoE4). ApoE, containing 299 residues, is a lipid-carrying protein that plays a crucial function in lipid homeostasis (Mahley et al., 1999). ApoE transports lipids in both the plasma and the central nervous system through its interaction with low-density lipoprotein receptors (Hatters et al., 2006). ApoE protein has three primary isoforms identified by the ApoE2, ApoE3, and ApoE4, which differ at positions 112 and 158 of the N-terminal domain. The ApoE2 isoform contains cysteine residues at both positions, ApoE3 has a cysteine at position 112 and an arginine at position 158, and the ApoE4 protein owns arginine residues at both positions (Chen et al., 2021). The three isoforms appear with varying frequency among the human population, indicating 8.45%, 77.9%, and 13.7%, respectively (Farrer, 1997). According to genome-wide association studies, the presence of two ApoE4 alleles is a significant genetic risk factor for late-onset Alzheimer’s disease (Corder, 1979).

As for the protein target hinted by the protein network analysis (e.g., ApoE4), a computational ligand-protein docking platform named Molsoft ICM Pro was then used to investigate the binding affinity between the protein target and each of 1.5 million chemical compounds from FDA-approved drugs and the ChemBridge database. Molsoft ICM outperforms a range of other methods for docking pose and affinity prediction. Molsoft ICM was evaluated with over 90% accuracy in flexible docking and covalent docking, which was better than other programs like Autodock, DOCK, FlexX, Gold, FITTED and MOE (Chilingaryan et al., 2021). ICM was ranked first place for docking pose and energy prediction in the drug design data resource (D3R) challenge for both 2017 and 2018 (Lam et al., 2019). The structures of chemical compounds with top binding affinity scores were further studied via statistical clustering analysis to identify the structures and interactions shared by those compounds for future drug design and experimental validation.

## 2 Materials and methods

**FIGURE 1:**
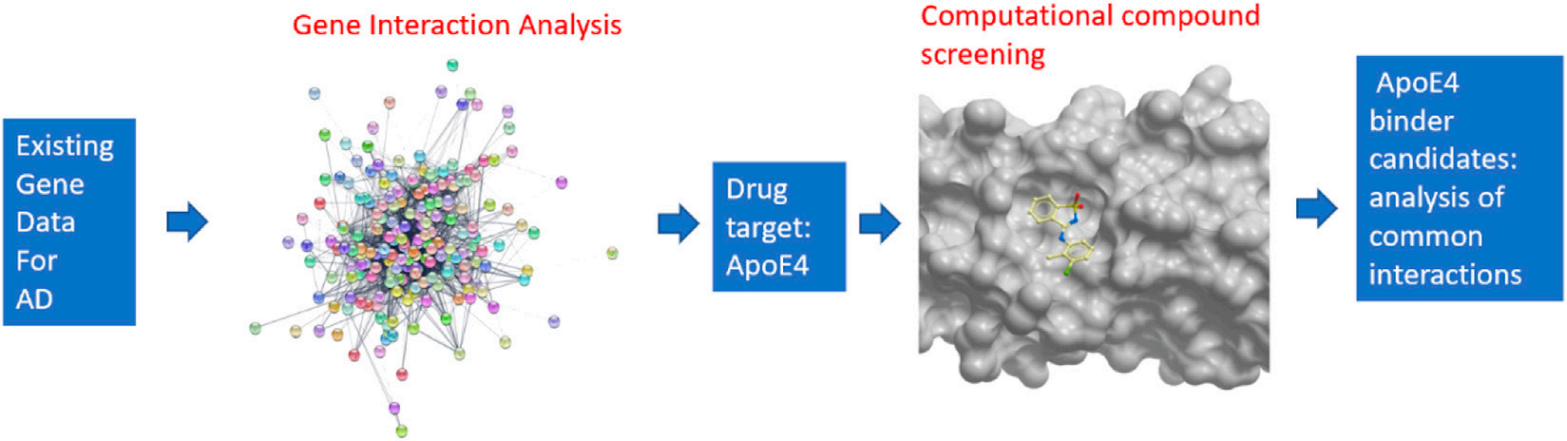
Overview of the pipeline for integrating systems biology and virtual compound screening to identify inhibitors for ApoE4.

An overview of the developed pipeline that integrates system biology and computational compound screening to identify potential drug candidates to treat Alzheimer’s disease is shown in Figure 1. In particular, a machine learning-based network analysis on genes related to Alzheimer’s disease was conducted. The proteins encoded by those genes were then connected in an interaction network so that the proteins with the largest interactions with others could be identified. Apolipoprotein E4 (ApoE4) turned out to be the top protein target from this study. Though a few fragments were co-crystalized with ApoE N-terminal domain (Petros, 2019), however these molecules suggest low binding affinity. It is hypothesized here that the binding of chemical compounds, which are more suitable as drug candidates than small fragments, may inhibit the function of ApoE4, thereby contribute to the combat of Alzheimer’s disease. On the basis of a validated crystal structure of ApoE4 (Petros, 2019), a computational compound screening platform was developed to evaluate the binding affinities of compounds from FDA-approved drugs and the ChemBridge database. The virtual screening platform consists of a ligand-protein docking program (i.e., Molsoft ICM Pro) to evaluate the binding affinities and an unsupervised learning method to identify the common structures shared by the compounds with good binding affinities with ApoE4. The interactions between those structures and residues in the binding pocket in ApoE4 were further analyzed to identify certain patterns conserved in the interactions.

### 2.1 The pipeline for the analysis of gene interaction network

**FIGURE 2:**
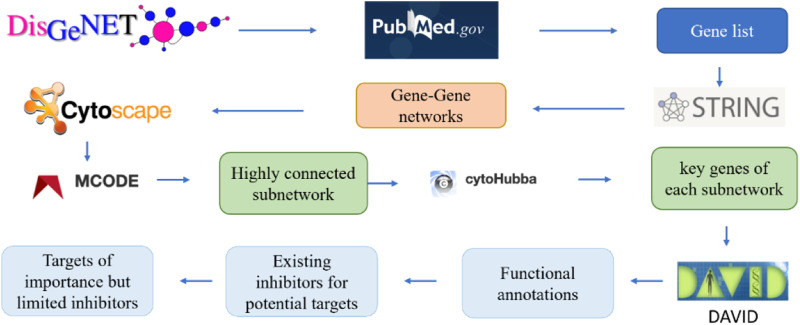
The pipeline to identify the key genes related to Alzheimer’s diseases via gene-gene network analysis.

Figure 2 illustrates the pipeline for building the interaction network of genes involved in the progression of Alzheimer’s disease. These genes were then ranked according to their interaction with other genes. Specifically, the key word “Alzheimer’s disease” was used in the DisGeNET database v6.0 (Barcelona, Spain), to search the relevant genes. The DisGeNET database returns a gene-disease-association (GDA) score for each gene (Piñero, 2017). A higher GDA score indicates a stronger association between the gene and Alzheimer’s disease. A GDA score of 0.1 was used in this work to narrow down the focus of a list of 3,397 Alzheimer’s disease-associated genes (Supplementary Table S1). The threshold of 0.1 maintained a manageable number of candidate genes (221 genes) for further analysis while still capturing potentially relevant gene-disease associations. All these genes were further validated for their association with Alzheimer’s disease in PubMed.gov by searching for each gene’s symbol along with the keywords “Alzheimer’s" or “Alzheimer’s disease” in the literature database. A systems-biology approach was employed to study gene-gene interactions in AD and identify the most significant genes. Specifically, the genes associated with Alzheimer’s disease, as determined by their GDA scores over 0.1, were input into the STRING program. This allowed for the generation of a protein-protein interaction network based on the proteins encoded by the selected genes. Subsequently, the protein interaction network was imported into the Cytoscape program to enable improved visualization and analysis. These genes were then clustered and ranked according to their interactions with other genes. To analyze the generated network, MCODE, a clustering algorithm, was used to discover densely connected proteins that may represent molecular complexes in huge protein-protein interaction networks. In this study, the generated networks were clustered using MCODE with parameters setting of the degree cutoff 2, a haircut option, and 0.47 minimum score. The degree cutoff value is the parameter that prevents nodes with fewer connections from being scored and included in a cluster. The highly interconnected proteins were grouped. However, when a cluster of nodes has been calculated and pulled out from the full network, some nodes might only have one interaction with the nodes in the cluster. The haircut option would eliminate these types of nodes from the established cluster. This parameter choice has been supported by previous research and is the default setting in the MCODE plugin for Cytoscape (Bader & Hogue, 2003; Brohee & van Helden, 2006). Furthermore, different degree cutoff values (1, 3, 4, and 5) were tested, and the results of the subnetworks remained consistent. The “minimum score” setting, also referred to as the node score cutoff, determines the minimum score a node must have to be included in a cluster, which would impact the size and number of subnetworks (Bader and Hogue, 2003). Therefore, different minimum scores other than 0.47 were tested to validate the consistency of top genes across different cutoff values. From the output of MCODE, highly linked nodes and their corresponding interactions were identified.

Additionally, the application cytoHubba was used to further rank the protein targets with the most interactions with other proteins in the subnetworks determined by MCODE with the MCC (Maximal Clique Centrality) algorithm. Earlier research indicated MCC as the method that was able to identify more important proteins than the other methods. The MCC algorithm was thus employed for this investigation (Chin et al., 2014). The top-ranked proteins may reveal potential drug targets to treat Alzheimer’s disease. The tool DAVID was implemented to investigate the functions of those proteins, which was followed by a thorough literature and patent review of existing inhibitors for each top-ranked protein. Finally, the top-ranked protein target with limited studies of associated compound binder was selected as the target for virtually screening small molecule inhibitors.

### 2.2 The crystal structure of the N terminal of ApoE4

While the detailed result of gene network analysis will be shown in the Results section, it turns out that ApoE4 is the top-ranked protein target with limited studies of associated compound binders. ApoE4 is indeed regarded as one of the greatest genetic risk factors for late-onset Alzheimer’s disease, and around 50% of people diagnosed with Alzheimer’s have the ApoE4 (Serrano-Pozo et al., 2021). In addition to ApoE4, Apolipoprotein E has two other alleles, which are ApoE 3 and ApoE 2 (Butterfield and Mattson, 2020). ApoE 3 is the most common Apolipoprotein E allele (i.e., 77.9% of alleles), followed by ApoE4 (13.7% of alleles) and ApoE 2 (8.4% of alleles). Different from ApoE4, reports show that ApoE2 protects against Alzheimer’s disease through both amyloid-beta (Aβ)-dependent and independent mechanisms (Ana-Caroline, 2011; Keeney et al., 2015; Li et al., 2020). The ApoE protein is made up of two domains (i.e., N-terminal and C-terminal) and 299 residues, and the three ApoE isoforms differ in two amino acid residues (i.e., 112 and 158) in the N-terminal. In particular, ApoE2 has C112 and C158, while ApoE4 has R112 and R158. This difference is reflected in the fact that the N-terminal domain of ApoE4 contains a receptor binding site while the N-terminal domain of ApoE2 does not. It is reported that ApoE alters the clearance of amyloid-β in an isoform-dependent manner (Ana-Caroline, 2011). Specifically, very-low-density-lipoprotein-receptor (VLDLR) and low-density-lipoprotein-receptor (LDLR) play an important role in cleaning amyloid-β through blood-brain-barrier (Yamazaki et al., 2019). This is impaired by ApoE4, as these receptors may bind to the N-terminal of ApoE4. It is reasonable to infer that the binding of chemical compounds to the N-terminal of ApoE4 may alter its function in regulating the clearance of amyloid-β.

**FIGURE 3:**
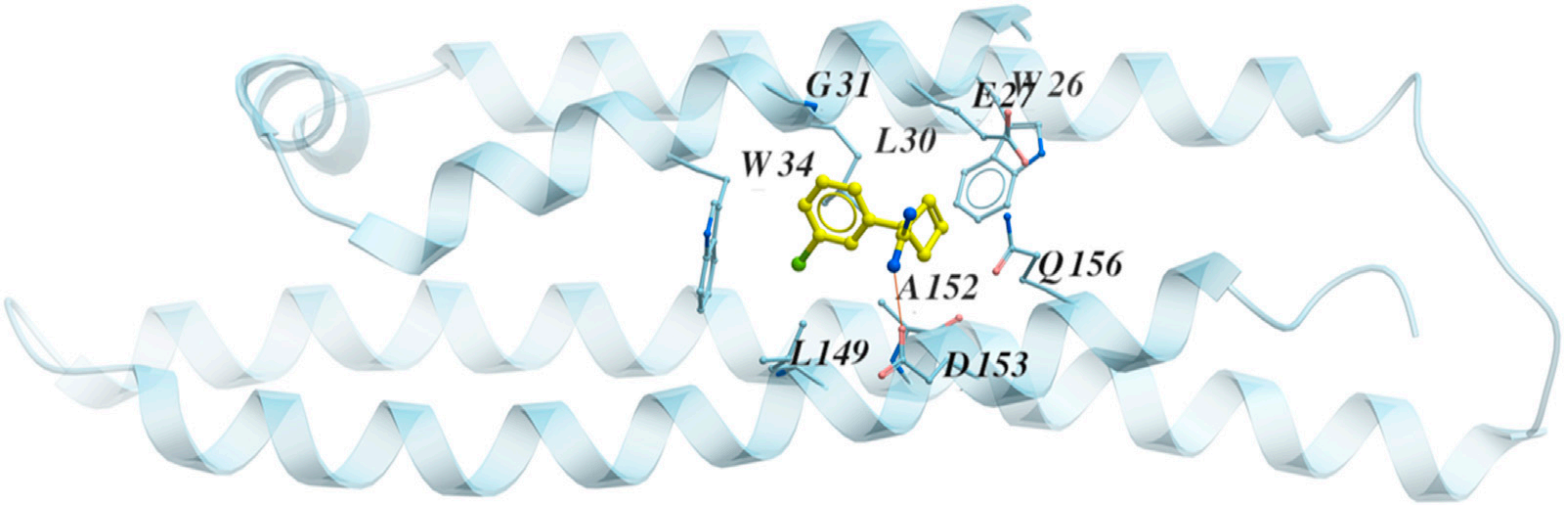
The structure of the N terminal of ApoE4 (blue) with the co-crystalized fragment (yellow) PDB code 6NCN. The residues around the binding pocket are shown with the fragment.

While the crystal structure of the full-sequence ApoE4 protein is not available, the N-terminal of ApoE4 (blue) was co-crystalized with a fragment (yellow) by a research group from Abbvie (North Chicago) (Petros, 2019), shown in Figure 3. The rotation of residues W26 and W34 constructs a hydrophobic pocket for binding (PDB code 6NCN). Experimental data from this group indicates that ApoE alleles show isoform-specific differences in ‘opening’ of the ApoE N-terminal domain helix bundle structure to facilitate a binding interaction of its hydrophobic interior with the lipid surface. Once the co-crystalized fragment binds to the hydrophobic pocket in the N-terminal of ApoE4, the physical properties of ApoE4 are changed, and ApoE4 would be more functional like ApoE3 and ApoE2 in liposome breakdown (Petros, 2019). Since the co-crystallized fragment is less than 200 Da, it is not a good drug candidate according to the Lipinski’s Rule of 5 (Lipinski et al., 1997; Lipinski et al., 2001; Lipinski et al., 2012; Walters, 2012). This motivates us to identify chemical compounds binding to the N-terminal domain of ApoE4.

### 2.3 Virtual screening of chemical compound databases to identify high-affinity ApoE4 binders

**FIGURE 4:**
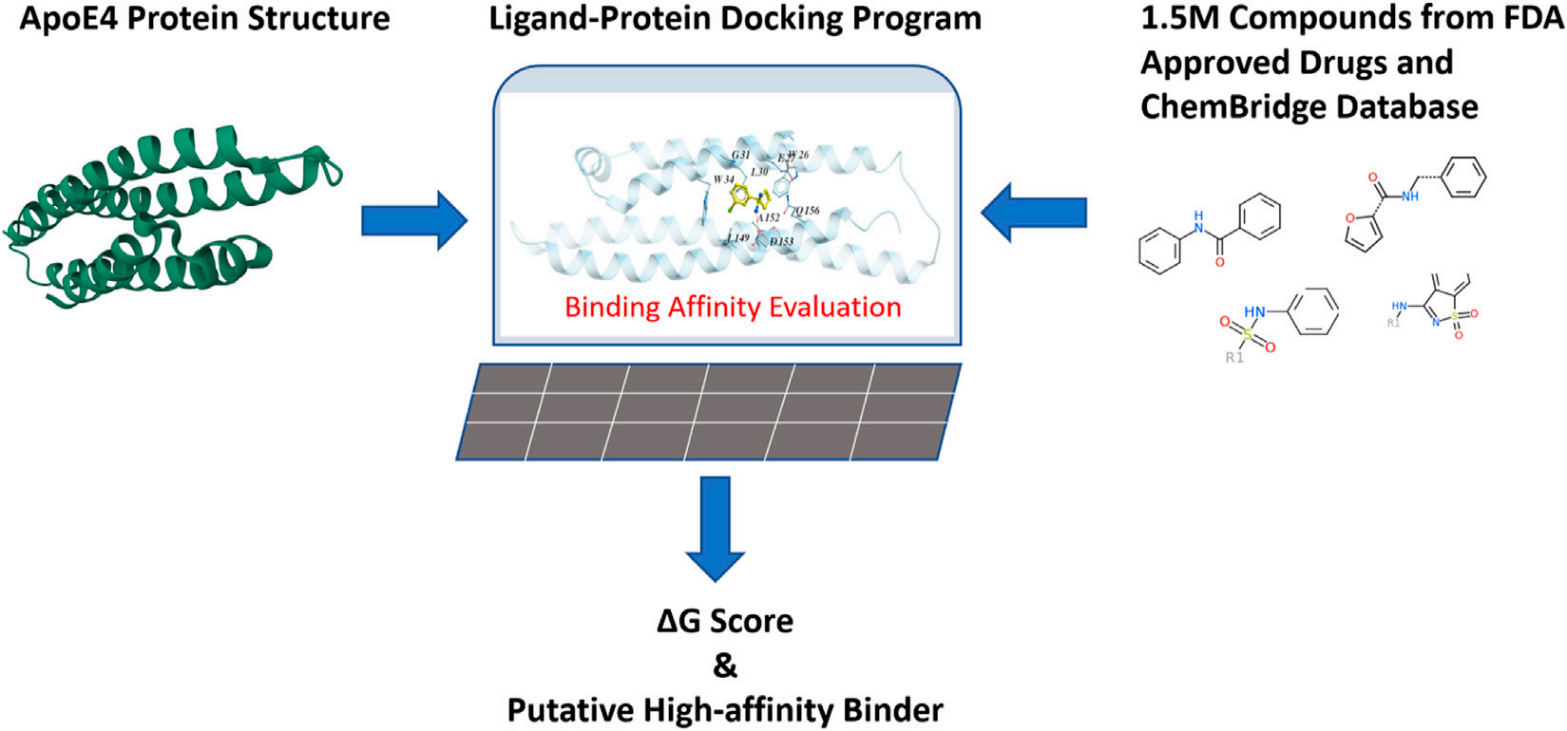
Virtual evaluation of 1.5 million chemical compounds from FDA-approved drugs and the ChemBridge database for their binding affinities with the binding pocket in the N-terminal of ApoE4.

Since it is costly and time-consuming to experimentally screen a large number of chemical compounds for their bindings to ApoE4, a computational approach was implemented in this work. The druggability of ApoE4 was first evaluated by Merck’s Drug-like density score (DLID) (Sheridan et al., 2010) to confirm that ApoE4 is suitable as a drug target. Figure 4 illustrates the general steps for virtual compound screening. The crystal structure of the N-terminal domain of ApoE4 from PDB (i.e., 6NCN) was input into the ligand-protein docking platform (i.e., Molsolf ICM Pro). The interaction between each chemical compound and the binding pocket in ApoE4 N-terminal was thoroughly evaluated to quantify the H-bond energy, van der Waals interaction energy, hydrophobic energy, desolvation energy, solvation electrostatic energy, internal molecular energy, loss of entropy, and number of atoms in the complex. A binding score that integrates the aforementioned interaction terms was determined to evaluate the binding affinity of each tested chemical compound to the N-terminal domain of ApoE4. Generally, a lower binding score indicates a lower the Gibbs energy of binding, which is correlated with a better binding affinity. Different ligand-protein docking programs use different algorithms to: 1) quantify the aforementioned interaction terms for potential binding postures of the compounds, and 2) determine the binding score. Molsolf ICM Pro is preferred in this work due to its better performance when compared to other platforms (Chilingaryan et al., 2021), (Lam et al., 2019), (Neves et al., 2012). The research team in this work has implemented Molsolf ICM Pro to successfully identify and experimentally validate several small molecule inhibitors against protein targets involved in antimicrobial resistance of foodborne pathogens (Zhang et al., 2020), (Zhang et al., 2022) and replication of SARS-CoV-2 (Zhai et al., 2021).

Around 1.5 million chemical compounds from the FDA-approved drugs and the ChemBridge database which contains drug-like chemicals were evaluated in this work. The ligand-protein docking program was conducted in a Dell workstation with 24 CPU cores and 192G memory. A compound library containing the structures of selected compounds was constructed and connected to the ligand-protein docking program so that the binding affinity between each compound and ApoE4 was evaluated (which took three to 10 seconds per compound), according to Eq. 1.

ΔG=ΔEHBond+ΔEVwlnt+ΔEHPhob+ΔESolv+ΔESolEl+ΔEIntl+TΔSSc+Qatom (1)

where Δ*E*HBond, Δ*E*Vwlnt, Δ*E*HPhob, Δ*E*Solv, Δ*E*SolEl, Δ*E*Intl, *T*Δ*SSc*, *Q*atom represent H-bond energy, van der Waals interaction energy, hydrophobic energy, desolvation energy, solvation electrostatic energy, Internal molecular energy, loss of entropy, number of atoms, respectively.

The binding score of the co-crystalized fragment with ApoE4 turns out to be −15 kcal/mol. Since the goal of this work is to identify compounds with better binding affinities than the co-crystalized fragment, the binding score of −20 kcal/mol was used to select compounds for further evaluation. This binding score has a margin of −5 kcal/mol from the one for the co-crystalized fragment to guarantee better binding affinities.

The 1,319 compounds were identified from the virtual screening and further evaluated for their druglike properties, toxicity, and ability to penetrate the blood brain barrier through ICM software. The ICM drug-likeness score was calculated using Molsoft’s internal model, which calculates chemical fingerprints of 5,000 marketed drugs from World Drug Index (positives) and 10,000 carefully selected non-drug compounds (negatives) (Kashgari et al., 2020). ICM Toxicity score (Toxscore) is determined approximately from one thousand SMARTS strings with known toxicity/reactivity that were gathered from diverse sources and calculated based on the number of present bioactive chemical fragments that were identified as structural alerts (Svetlov, 2018). The Blood Brain Barrier prediction score has been calculated by an algorithm, designated “BBB Score”, composed of stepwise and polynomial piecewise functions (Gupta et al., 2019). According to these existing literatures, the compounds need to meet the specific features for further considerations: an ICM drug-likeness score of [-1, 1], a toxicity score of [0, 1], a blood brain barrier score of (Alzheimer’s Association, 2020; Li, 2018). Therefore, the 312 compounds meeting all these conditions specific features would be candidates of ApoE4 binders.

### 2.4 Investigation of common structure and essential function groups of identified ApoE4 binders

**FIGURE 5:**
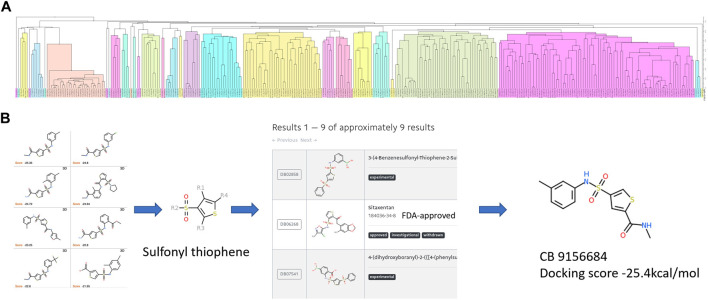
The procedure to identify the common structures with strong binding affinities with the binding pocket in the N-terminal of ApoE4: **(A)** hierarchical clustering to separate chemical compounds into different groups according to their chemical structures; **(B)** common structures identified for individual groups and validated in the Drugbank database. Sulfonyl thiophene was identified as the common structure for the group. Nine approved or experimental drugs were found in Drugbank database with sulfonyl thiophene. The ChemBridge compound (CB915k6684) had the lowest binding score in the group of compounds.

The candidates of ApoE4 binders selected from Section 2.3 were further analyzed to identify the common structures and essential functional groups shared by those compounds. Chemical clustering methods based on substructure similarity were applied to identify the common structures and essential functional groups which contribute to ligand-protein interactions. The 2D chemical structure information was used to cluster the compounds into different groups using unweighted pair group method with arithmetic mean (UPGMA) clustering method. Figure 5A shows the clustering of 312 selected compounds into 109 groups, which are represented in different colors. The details of the 312 compounds can be found in Supplementary Table S2. The number of groups was determined by the elbow method that depends on the change of the cumulative variance of all groups (Thorndike, 1953). The 3D binding conformations of compounds in each group were aligned and compared to find common substructures. The common structure of the compounds in each group was then identified and evaluated on the basis of the Drugbank database. The common structures that are not related to special functions, such as carbon double bond and benzene ring, were generally not specific to known drugs in the Drugbank database. Figure 5B illustrates the structure of sulfonyl thiophene was identified from a group of compounds. It was input the Drugbank database, and 9 drugs with clinical experimental evaluation or approved by FDA were found. For example, sitaxentan is one of the nine drugs containing sulfonyl thiophene. It has been approved by FDA for the treatment of the treatment of pulmonary arterial hypertension. The chemical compound with the lowest binding score was selected for each group to investigate the common interaction between the group compounds and the binding pocket in the N-terminal of ApoE4. Compound ChemBrige 9156684 (CB9156684) was selected to represent the group to study the role of sulfonyl thiophene in binding to ApoE4 in this study. The docking pose of compounds with specific common structures was manually checked to find any hydrogen bonds between the functional groups and protein residues. If a hydrogen bond interaction was formed between the same functional groups and same residues among various compounds, the interaction between the functional groups and residues was regarded as a conserved interaction. Conserved interactions between functional groups of ligands and protein residues are helpful for drug development. Core compounds were selected as representatives to illustrate the interactions between specific functional groups and residues of the binding pocket of ApoE4.

## 3 Results

### 3.1 Identification of protein targets for Alzheimer’s disease through gene interaction network analysis

**FIGURE 6:**
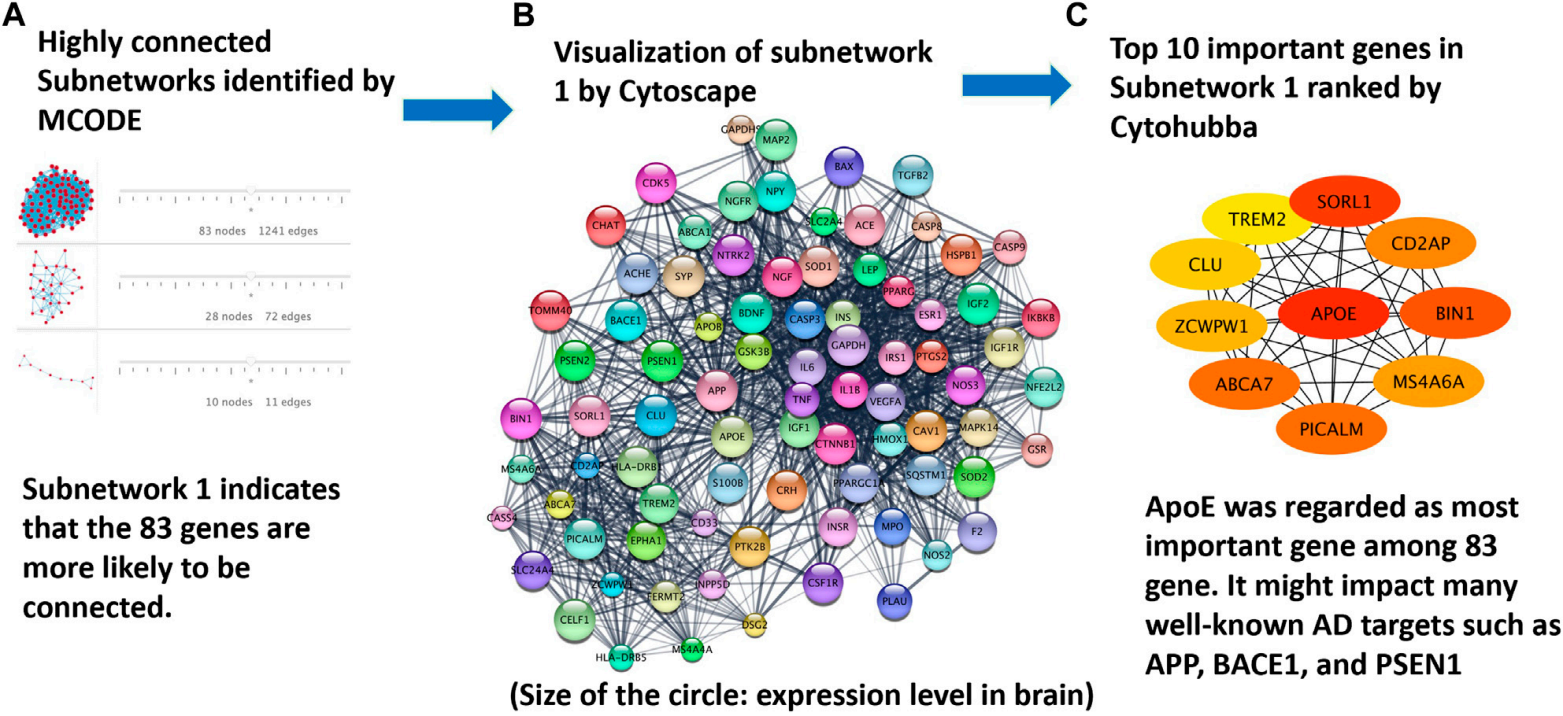
Protein targets identified from the analysis of protein interaction networks: **(A)** highly-connected subnetworks identified by the program MCODE; **(B)** the subnetwork of proteins visualized in Cytoscape to further study the interaction of these proteins; and **(C)** top proteins ranked by Cytohubba on the basis of their interactions with other proteins. ApoE4 was ranked the top one protein target for Alzheimer’s disease treatment.

The approach for protein interaction network analysis shown in the Methods section was implemented to the 208 genes identified and validated from DisGeNET database. Specifically, the key word “Alzheimer’s disease” was used in the DisGeNET database, which returned 3,397 genes, as shown in Supplementary Table S1. A GDA score of 0.1 was used in this work to narrow down the focus to 221 genes. Subsequently, 208 genes can be identified as valid nodes by STRING to build a network. Figure 6 shows the highly connected subnetworks generated by MCODE that contain more than 10 nodes (i.e., proteins). Each subnetwork was further explored for the protein-protein interactions. The subnetwork with 83 nodes was selected and zoomed in Figure 6B. Different proteins are presented in different colors, with their sizes indicating their expression level in the brain. This subnetwork was selected because it contains certain proteins that are known for their involvement in Alzheimer’s disease progression. For example, APP, PSEN1, BACE1, and PSEN2 are all in this subnetwork. It is reasonable to hypothesize that the top drug target for Alzheimer’s disease should interact with or influence these proteins through known mechanisms for regulating Alzheimer’s disease. The program Cytohubba in Cytoscape was further used to rank the proteins in the subnetwork in Figure 6A to identify the top 10 targets for treating Alzheimer’s disease (Figure 6C). The redness indicates the importance of the compounds. ApoE was ranked as the top target, followed by SORL1, BIN1, ABCA7, PICALM, CD2AP, MS4A6A, ZCWPW1, CLU, and TREM2. Furthermore, irrespective of threshold setting of the minimum score, ApoE consistently emerged as a top-ranking gene in the subnetworks. This suggests that ApoE4 is a core component of these subnetworks, reinforcing its importance in the context of Alzheimer’s disease. Additionally, it is interesting to find that all these proteins have been extensively studied for their involvement in the progression of Alzheimer’s disease. Particularly, ApoE, especially ApoE4, is involved in carrying cholesterol and other types of fat in the brain (Butterfield and Mattson, 2020). SORL1 protein, also known as SORL1 or LR 11, shunts amyloid precursor protein (APP) into non-amyloidogenic processing pathways, and loss of SORL1 leads to higher amyloid-β levels in cell culture experiments (Yin et al., 2015). The overexpression of BIN 1 (i.e., the Bridging Integrator 1) was found to result in an increase in the size of early endosomes and neurodegeneration and contribute to early-endosome size deregulation, which is an early pathophysiological hallmark of AD pathology (Lambert, 2022). ABCA7 (i.e., ATP-binding cassette transporter A7) variants are regarded as susceptibility loci for late-onset Alzheimer’s disease, as ABCA7 deficiency exacerbates amyloid-β pathology and it is also involved in the microglial amyloid-β clearance pathway (Aikawa et al., 2018). PICALM, an accessory protein in the endocytic pathway, was identified in one of the first large-scale genome-wide association studies (GWAS) for late-onset Alzheimer’s disease. It is reported to affect the internalization of APP and thus the production of amyloid-β (Harold, 2009). CD2AP (i.e., CD2 associated protein) plays a role in maintaining early endosome morphology and the traffic between early and late endosomes (Tao et al., 2019), in regulating amyloid-β generation by a neuron-specific polarization of amyloid-β in dendritic early endosomes (Ubelmann, 2017), and in affecting APP and BACE1 sorting in early endosomes by distinct mechanisms (Sun and Roy, 2018). MS4A6A (i.e., membrane-spanning 4-domains, subfamily A, member 6A), susceptibility loci of Alzheimer’s disease, was found to be related to the volume loss of middle temporal, precuneus, and entorhinal in the brain (Ma, 2016). ZCWPW1 (i.e., Zinc Finger CW-Type and PWWP Domain Containing 1), a histone modification reader that is involved in epigenetic regulation, was reported for strong association with late-onset Alzheimer’s disease in several reports (Rosenthal and Kamboh, 2014; Gao, 2016; Karch, 2016; Küçükali, 2021). CLU (i.e., clusterin) could bind amyloid-β peptides and prevent fibril formation, a hallmark of AD pathology (Wojtas, 2020). TREM2 (i.e., Triggering receptor expressed on myeloid cells 2) induces microglial anti-inflammatory activation in AD-related conditions, and it is regarded as a potential target for the prevention and treatment of Alzheimer’s disease (Carmona et al., 2018), (Ulrich et al., 2017).

While the 10 proteins shown in Figure 6B are all heavily involved in the cause or progression of Alzheimer’s disease, ApoE4 was selected as the protein target for this study as it was ranked as a top genetic risk factor by this study and other studies (Vassar, 2017; Fernandez et al., 2019; Safieh et al., 2019). In addition, few drugs have been investigated or identified for ApoE4. Finally, the crystal structure of the binding pocket in ApoE4 has been published (Petros, 2019).

### 3.2 Virtual screening of chemical compounds to bind ApoE4 N-terminal domain

**FIGURE 7:**
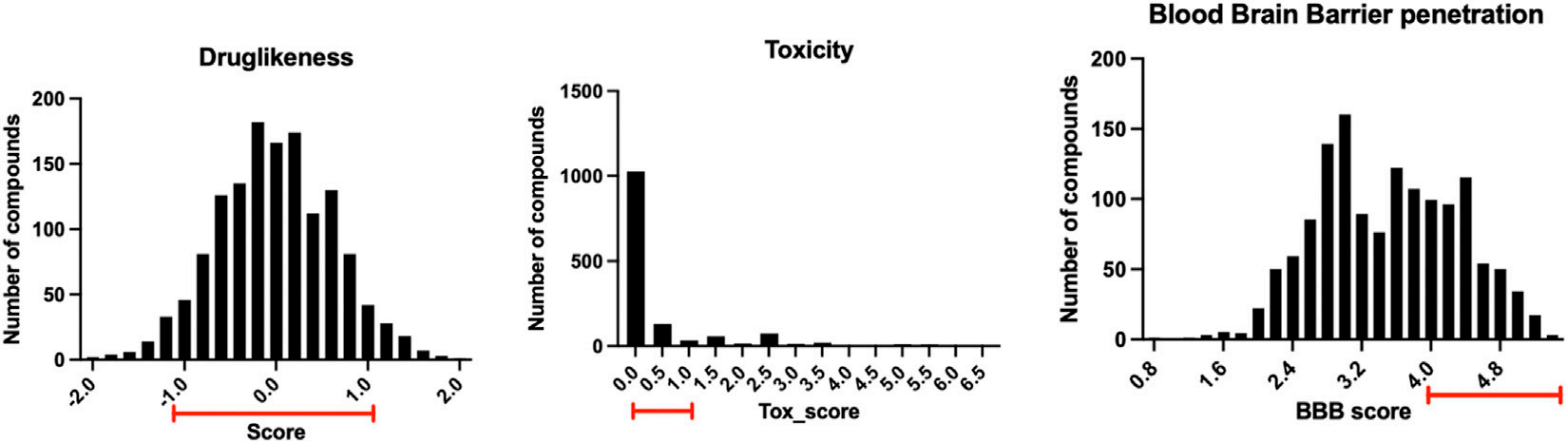
The distribution of the 1,391 compounds selected by the ligand-protein docking program in their druglike properties, toxicity, and ability to penetrate blood brain barrier.

The 1.5 million chemical compounds from the FDA approved drugs and ChemBridge database were evaluated by the ligand-protein docking program shown in Figure 4. There were 1,391 compounds with binding scores less than −20 kcal/mol (less than the score of −15 kcal/mol for the co-crystalized fragment). These compounds were further evaluated by their druglike properties, toxicity score, and the ability to penetrate the blood brain barrier (BBB score) with the conditions specified in the Methods section. The compounds that satisfy each condition are marked in the histograms shown in Figure 7. While most compounds have satisfied druglike and toxic properties, only one-third of the compounds might penetrate the blood brain barrier. A total of 312 chemical compounds were finally selected as druggable candidates for binding ApoE4 for Alzheimer’s disease intervention.

### 3.3 The common structures of putative ApoE4 binders

**FIGURE 8:**
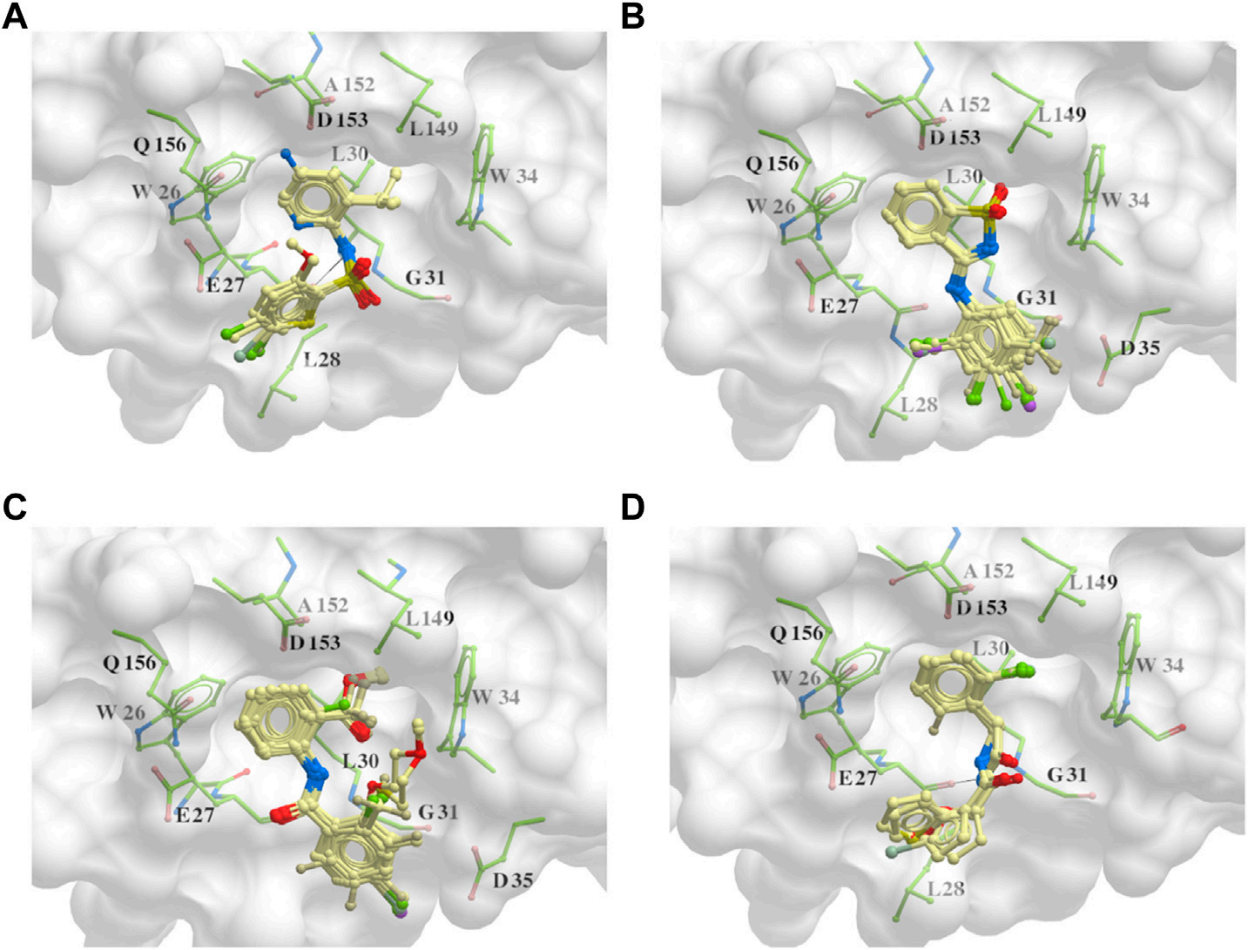
Common structures extracted from the identified ApoE4 binders by the clustering of 2D structures and the alignment of 3D conformations: sulfon-amine-benzene **(A)**, 1,2-benzisothiazol-3-amine 1,1-dioxide **(B)**, *N*-phenylbenzamide **(C)**, furan-amino-benzene **(D)** were obtained from four large groups of candidates. The putative ApoE4 binders (yellow), amino acid residues of ApoE4 protein (green), and surface of ligand binding pocket (transparent white) were displayed using ICM.

The 312 putative ApoE4 binders indicating various docking conformations were clustered into 109 different groups, results of which were shown in Supplementary Table S2. Four large groups containing more than ten compounds indicated certain common structures. The compounds sharing similar structures would bind to the ApoE4 protein with similar 3D docking poses. This suggests that those similar structures shared by various compounds fit well into the binding pocket. The four common structures, which include sulfonamide-benzene, 1,2-benzisothiazol-3-amine 1,1-dioxide, *N*-phenylbenzamide, furan-amino-benzene, were obtained by 3D conformation alignment, as shown in Figure 8. Such four scaffolds fit the binding pocket of the co-crystalized fragment and extend to sub pocket p1 or p2, shown in Supplementary Figure S1. Several molecular interactions were observed conserved among the common structures, which were listed in Table 1. The sulfon-amine-benzene structure might occupy the main ligand binding pocket by the benzene or pyridine group via hydrophobic interactions or VWD forces with the residues (shown in Figure 8A; Supplementary Figure S2). The NH group might form hydrogen bond with residues E27, which can further enhance the binding affinity. Substitute alkyl group on benzene could extend the interaction with residues L149 and W34. Additional six or five member rings linked to sulfonamide would bind to sub pocket p1 formed by E27 and L28. The common structure of 1,2-benzisothiazol-3-amine 1,1-dioxide might fully occupy the main binding pocket, shown in Figure 8B; Supplementary Figure S3. The benzisothiazol group might form hydrophobic interactions or anion-pi interactions with the main binding pocket, in which the dioxide group would contact W34 by VDW. The benzene rings might undergo functionalization with different substituents, such as alkyl or chloro groups. The structure *N*-phenylbenzamide might fit the main pocket and the sub pocket 2 by the two benzene rings, shown in Figure 8C; Supplementary Figure S4. Nonetheless, the furan group of the common structure 4 might prefer binding to the sub pocket p1, as shown in Figure 8D; Supplementary Figure S5. While these common structures dominate the interactions between the identified compounds with the residues in the binding pocket of ApoE4, other functional groups like carboxylic acid, ketone, amine, amide, and hydroxy also contribute to the binding (as shown in Table 1). These common structures, along with their interactions with residues in the binding pocket of ApoE4, pave the way for future compound optimization. For example, the interactions between the functional groups of ligands and the listed residues in Table 1 can serve as a starting point for ApoE4 drug development.

**TABLE 1:** Summary of the common interactions between identified compounds and ApoE4 protein.

Chemical structure	Structure name	Predicted interactions with individual residues
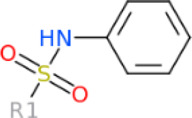	Sulfon-amine-benzene	Hydrogen bond (HB): E27, Hydrophobic: A152, W26 VDW: D153
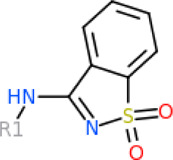	1,2-benzisothiazol-3-amine 1,1-dioxide	Cation/Anion-π interactions: Q156, D153 Hydrophobic: E27, L30, G31, W26, A152, L149 VDW: W34
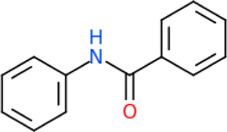	*N*-phenylbenzamide	Cation/Anion-π interactions: Q156 Hydrophobic: L30, L149, A152 VDW: D153
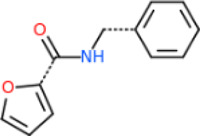	Furan-amide-benzene	HB: E27 Cation/Anion-π interactions: D153, Q156 Hydrophobic: W26, L28, L30, L149
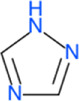	1,2,4-triazole	HB: D153, D53
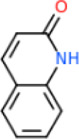	Quinolin-2(1H)-one	HB: Q156
-COOH	Carboxylic acid	HB: W34, R38
-CO-	Ketone	HB: W34, R38
-O-	Ether	HB: R38
-NH-CO-	Amide	HB: E27
-NH, -NH2	Amine	HB: W34, R38, E27
-OH	Hydroxy	HB: E27, D153

**FIGURE 9:**
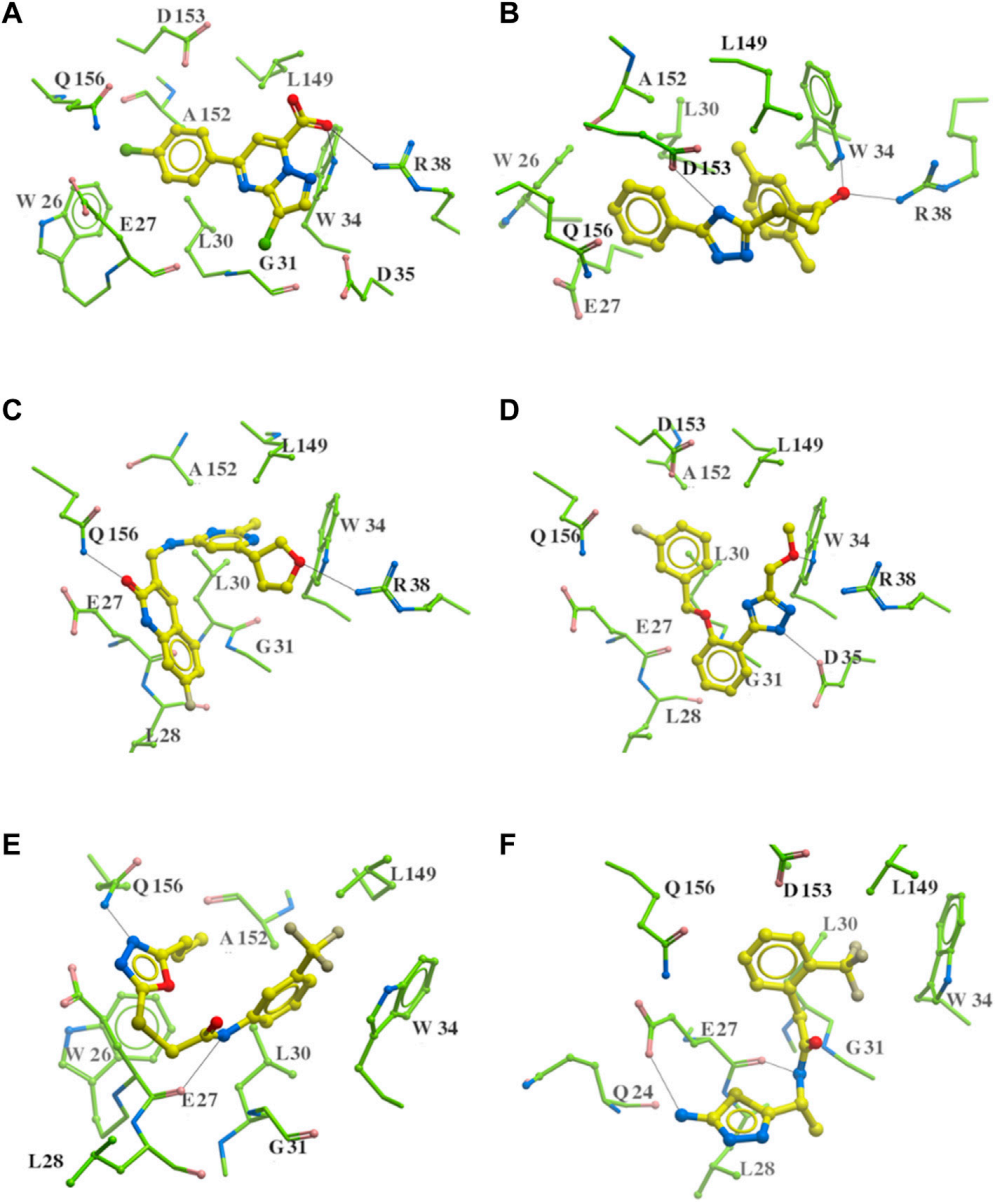
Six putative ApoE4 binders **(A–F)** present interactions identified conserved between ligand functional groups and ApoE4 residues. The hydrogen bonds formed by the compounds (yellow) and protein residues (green) were labelled in black solid line.

As highlighted in Figure 8; Table 1, the hydrogen bonds play an important role in the interactions between the common structures and ApoE4 residues. The 312 identified small molecules contain a variety of hydrogen bond donor or acceptor functional groups. In particular, the compounds are more likely to form hydrogen bonds with polar residues around the ligand binding pocket, for instance, residues W34, R38, E27, D35, D153, and Q156. To further illustrate this, six compounds were displayed as examples in Figure 9. The carboxylic acid group of compounds would form hydrogen bonds with both residues W34 and R38 (e.g., Compound a in Figure 9A). Compound b not only binds to residues R38 and W34 with the ketone functional group but also forms a hydrogen bond (HB) with D153 through the triazole group. Compound c binds to a HB donor, i.e., residue Q156, by the quinolin-2(1H)-one group and to R38 residues by an ether group. The triazole group of compound d and residues D35 might also have hydrogen bonds in between. As shown for Compounds e and f in Figures 9E, F, the amine and amide groups might bind to residues E27 by one or more hydrogen bonds. In short, some hydrogen bonds can be found conserved via a careful analysis of ligand-protein interactions. Carboxylic acid, ketone, amide, and ether might function as HB acceptors and bind to residues W34 and R38. Amine and amide groups acting as HB donors form hydrogen bonds with residues E27. Since these interactions were conserved in multiple compounds, they may be retained in other ligands with similar functional groups when they bind to ApoE4. Due to the limitation of hardware, the conformational change of every compound was limited during the virtual screening. The aforementioned functional groups may make directions of screening of large make-on-demanded libraries which might contain billions of compounds, and thus would reduce the simulation time.

## 4 Discussion

### 4.1 The identified drug target for Alzheimer’s disease

A systems-biology pipeline was developed in this work to identify potential protein targets to regulate the progression of Alzheimer’s disease. ApoE4 turned out as the top protein target. Interestingly, previous studies also attempted to prove ApoE4 as a protein that could increase the risk of Alzheimer’s disease. In an ApoE inducible mouse model, the ApoE4 isoform indicated a correlation with amyloid pathology, which impairs Aβ clearance and accelerates its aggregation during the early seeding stage of amyloid accumulation (Liu, 2017). Another mouse model of tauopathy demonstrated that ApoE4 affected tau pathogenesis, caused neuroinflammation, and exacerbated tau-mediated neurodegeneration (Shi, 2017). It is reported that the binding of the co-crystalized fragment to a hydrophobic pocket in ApoE4 may change the physical properties of ApoE4 and manipulate ApoE4 activity (Petros, 2019). The binding may make ApoE4 function like other Apolipoprotein E alleles (i.e., ApoE3 and ApoE2) in liposome breakdown. ApoE3 and ApoE2 are reported to have neutral and protective effects on the progression of Alzheimer’s disease, respectively (Huang et al., 2019). Although ApoE4 could be a valuable treatment target of Alzheimer’s disease, very limited study had used it as a target to develop new therapeutic methods of Alzheimer’s disease. This work thus represents the first systematic evaluation of ApoE4 as the target for identifying small molecules for Alzheimer’s disease intervention. In addition to ApoE4, other potential protein targets listed in Figure 6 can be further investigation for their involvement in the progression of Alzheimer’s diseases in the future. Besides, top 20 genes with high GDA scores, including ApoE, were further investigated for their involvement in Alzheimer’s disease through a literature review. The results have been detailed in Supplementary Figure S6.

### 4.2 Common structures of potential ApoE4 binder compounds

Since there are millions of chemical compounds, it is time consuming and costly to test each compound in experiments for its binding affinity with ApoE4. Different from the conventional drug discovery method, a computer-aided drug discovery and design method was developed in this work to discover compounds that could be used as binders of ApoE4. While it is meaningful to evaluate the 312 putative ApoE4 binders in experiments, it is challenging to obtain purified ApoE4 whole protein along with its activity assay at current stage. Instead, this work is focused on studying the common structures of the 312 identified compounds, as these functional groups may be used for further compound optimization.

Four common structures, i.e., sulfonamide-benzene, 1,2-benzisothiazol-3-amine 1,1-dioxide, *N*-phenylbenzamide and furan-amino-benzene, were identified via hierarchical clustering of compound chemical structures. The sulfonamide-benzene structure facilitates the binding to ApoE via hydrophobic interactions or VWD forces between the benzene or pyridine group with the residues (i.e., residuals L30, A152, W26, W34, L149 for hydrophobic interaction; residuals D153, Q156, G31 for VWD forces). The structure of 1,2-benzisothiazol-3-amine 1,1-dioxide shows hydrophobic interactions or anion-pi interactions with the binding pocket, especially with residuals L30, W26, Q156, D153, A152, L149 for hydrophobic interactions) and with residual W34 (via the SO_2_ group) for VDW. N-phenylbenzamide might fit the main pocket and the sub pocket 2 by the two benzene rings via the hydrophobic interaction with residuals A152, L30, G31. The furan-amino-benzene structure enables its binding to the sub pocket p1. The interactions between these structures shared by multiple compounds and residuals in ApoE4 can provide guidance to further optimize compound structures to obtain better binding affinities. This demonstrates that computational approaches are capable of studying detailed ligand-residual interactions that are challenging, costly, or time-consuming to experiment with.

### 4.3 Limitations and future work

The ligand-protein docking program in this work was based on the crystal structure of the N-terminal of ApoE4 from existing experimental data (Petros, 2019). The crystal structure of the C-terminal of ApoE4 is not available yet. One solution to address this is using AlphaFold, an AI system developed by DeepMind to predict the 3D structure of a protein from its amino acid sequence, to predict the crystal structure of ApoE4 with both N-terminal and C-terminal domains. This is the next direction for us to pursue before the crystal structure of ApoE4 is determined from experiment. The compounds along the discovered common structures will be further evaluated by the updated crystal structure of ApoE4.

This work mainly aims to identify compounds and common structures for further optimization of compounds for binding ApoE4. While experimental validation was planned and tried, no protein-based assay kit has been designed for ApoE4 yet. This is like the situation for the complete crystal structure of ApoE4. One approach to address this issue is to use mouse models with different ApoE alleles to evaluate the compounds and common structures discovered in this work. Researchers can use induced pluripotent stem cells (iPSC) to grow human neurons in a dish now (Lagomarsino, 2021). It may be possible to grow neurons with microglia and astrocytes in a dish to mimic the brain in the future. This may enable the testing of the ApoE4 binders in a dish.

Since ApoE4 is hinted by the literature as a genetic risk factor for Alzheimer’s disease, this work was mainly focused on identifying binders to ApoE4. The ApoE2 isoform contains cysteine residues at both positions, ApoE3 has a cysteine at position 112 and an arginine at position 158, and the ApoE4 protein owns arginine residues at both positions (Chen et al., 2021). This is an interesting direction to further study whether the identified compounds would be selective to ApoE4 rather than ApoE2 and ApoE3.

The dynamic system modeling formalism, such as The Cell Collective, would be helpful to investigate the complex feedback mechanisms and dynamic behavior of the protein-protein interaction network in Alzheimer’s disease. For example, molecular regulation with feedback circuits may be presented in Boolean networks (Azpeitia, 2017). In order to build and validate the dynamic models, gene regulation data over time is needed for Alzheimer’s disease. While the dynamic data is note complete for all AD genes in the DisGeNET, the dynamic system modeling formalism is an interesting direction for future investigations.

## 5 Conclusion

Alzheimer’s disease has become a major public health issue. After decades of extensive research, only seven drugs have been approved by the FDA to treat Alzheimer’s disease. One reason for this is that various genes, reactions, and pathways are involved in regulating the progression of Alzheimer’s disease. This work conducted a comprehensive analysis of genes from existing clinical database and identified ApoE4 as the top protein target to intervene Alzheimer’s diseases. Existing data indicated the binding of fragments to ApoE4 may change its function as the greatest risk factor of Alzheimer’s disease. On the basis of the crystal structure of ApoE4 N-terminal, a computational pipeline was developed to screen 1.5 million compounds from FDA approved drugs and ChemBridge database for their binding affinities with ApoE4. Totally 1,391 compounds were identified with better binding affinities than the co-crystalized fragment, and 312 of them have met the required druglike properties, low toxicity, and ability to penetrate blood brain barrier. Statistical analysis of the 312 compound structures indicated that the compounds with good binding affinities generally have the structures like sulfon-amine-benzene, 1,2-benzisothiazol-3-amine 1,1-dioxide, N-phenylbenzamide, and furan-amino-benzene. The common interactions between the 312 compounds and residues in ApoE4 were thoroughly analyzed. It turns out that the residues E27, W34, R38, D53, D153, and Q156 in the N terminal of ApoE4 play an important role in forming hydrogen bonds with compounds. Hydrophobic interactions are found between compounds with the following residues W26, E27, L28, L30, G31, L149, and A152. The four common compound structures and the interactions with the aforementioned residues can serve as starting points for future compound optimization to identify ApoE4 binders for potential Alzheimer’s disease intervention.

## Data Availability

The original contributions presented in the study are included in the article/Supplementary Materials, further inquiries can be directed to the corresponding author.
