# Supplementary material for: A computational framework for identifying chemical compounds to bind Apolipoprotein E4 for Alzheimer’s disease intervention
Source: Front Syst Biol. 2023 Jun 14;3:1188430. doi: 10.3389/fsysb.2023.1188430 (PMC12341960; doi:10.3389/fsysb.2023.1188430)
Supplement: Supplementary file 3 [file DataSheet1.docx]

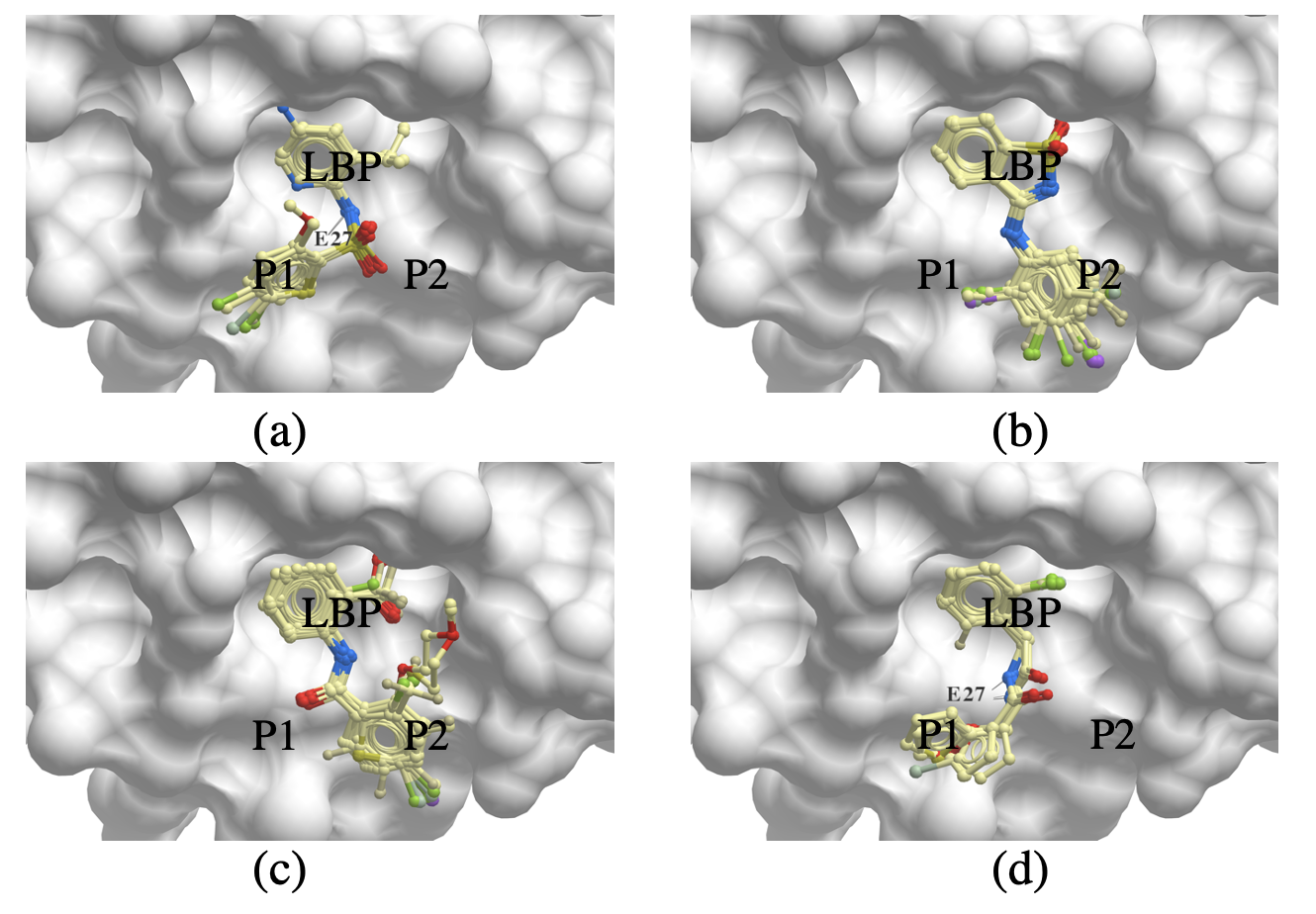


Supplement Figure1. Common structures extracted from the identified ApoE4 binders: sulfon-amine-benzene (a), 1,2-benzisothiazol-3-amine 1,1-dioxide (b), N-phenylbenzamide (c), furan-amino-benzene (d) were obtained from four large groups of candidates. The putative ApoE4 binders (yellow) and surface of ApoE4 protein (white) was displayed using ICM. Crystal ligand binding pocket (LBP), sub pocket 1 (P1), and sub pocket 2 (P2) were labelled.


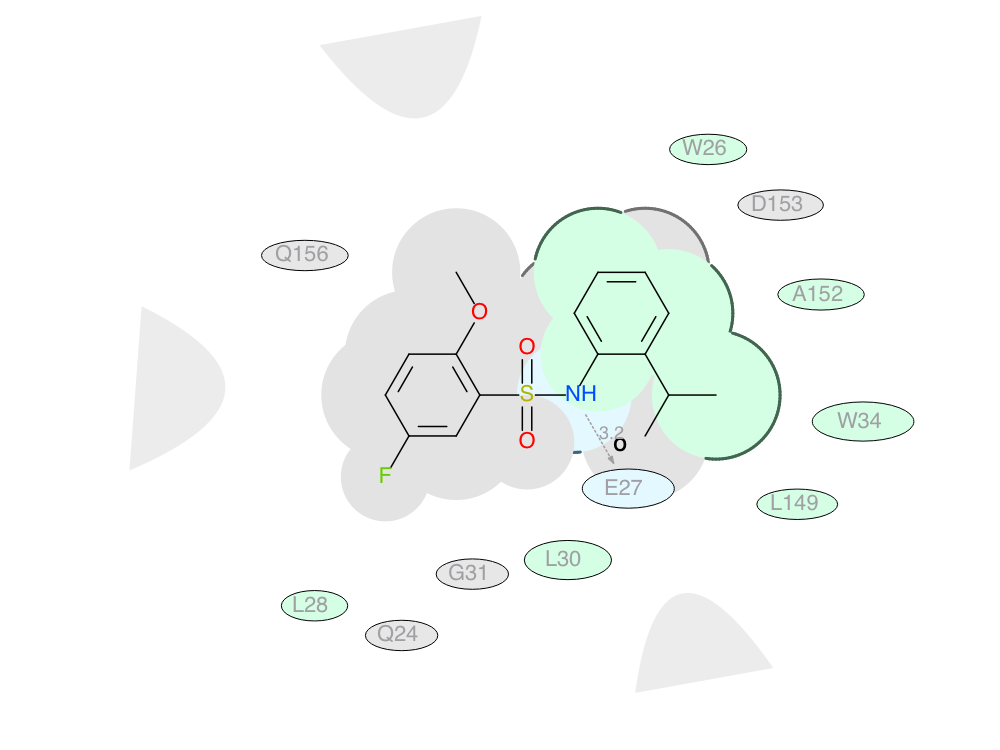


Supplement Figure2. Sulfon-amine-benzene (common structure 1) might occupy the main ligand binding pocket by the benzene group via hydrophobic interactions or VWD forces with the residues.The 2D illustration of the interaction between sulfon-amine-benzene and ApoE4. The image is annotated with hydrogen bonds and interacting residues. The residue interaction surface and proximity are represented by the size of the residue label and distance, respectively. Grey parabolas and broken thick lines indicate solvent accessible regions and the ligand is shaded by property. Here are the meanings of colors, lines, and sizes shown in the 2D interaction diagram (same in supplement figure 3, 4 and 5): 1) Green shading represents hydrophobic region; 2) Blue shading represents hydrogen bond acceptor; 3) White dashed arrows represent hydrogen bonds; 4) Grey parabolas represent accessible surface for large areas; 5) Broken thick line around ligand shape indicates accessible surface; 6) Size of residue ellipse represents the strength of the contact; 7) 2D distance between residue label and ligand represents proximity.


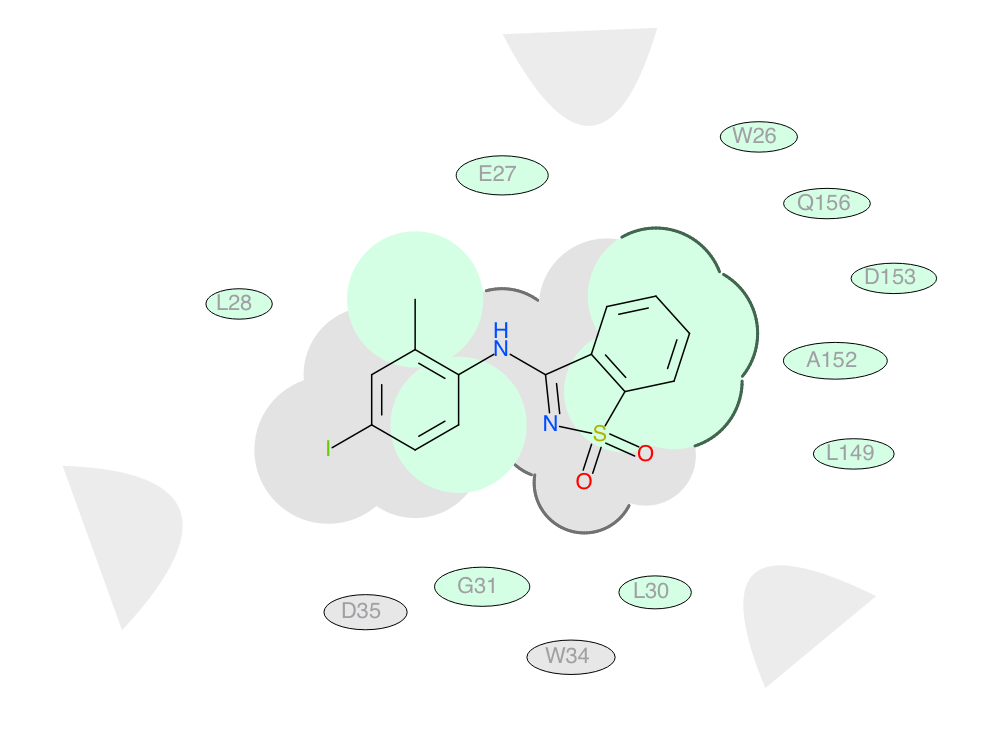
 Supplement Figure 3. 1,2-benzisothiazol-3-amine 1,1-dioxide (Common structure 2) might fully occupy the main binding pocket


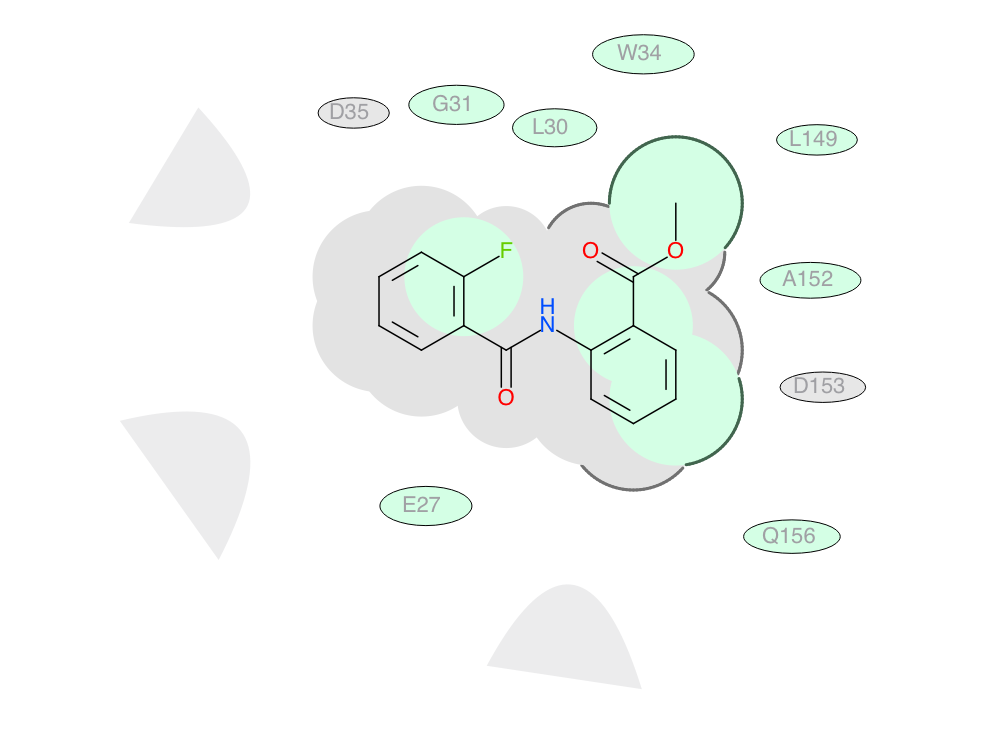


Supplement Figure 4. *N*-phenylbenzamide (Common structure 3) might fit the main pocket and the sub pocket 2.


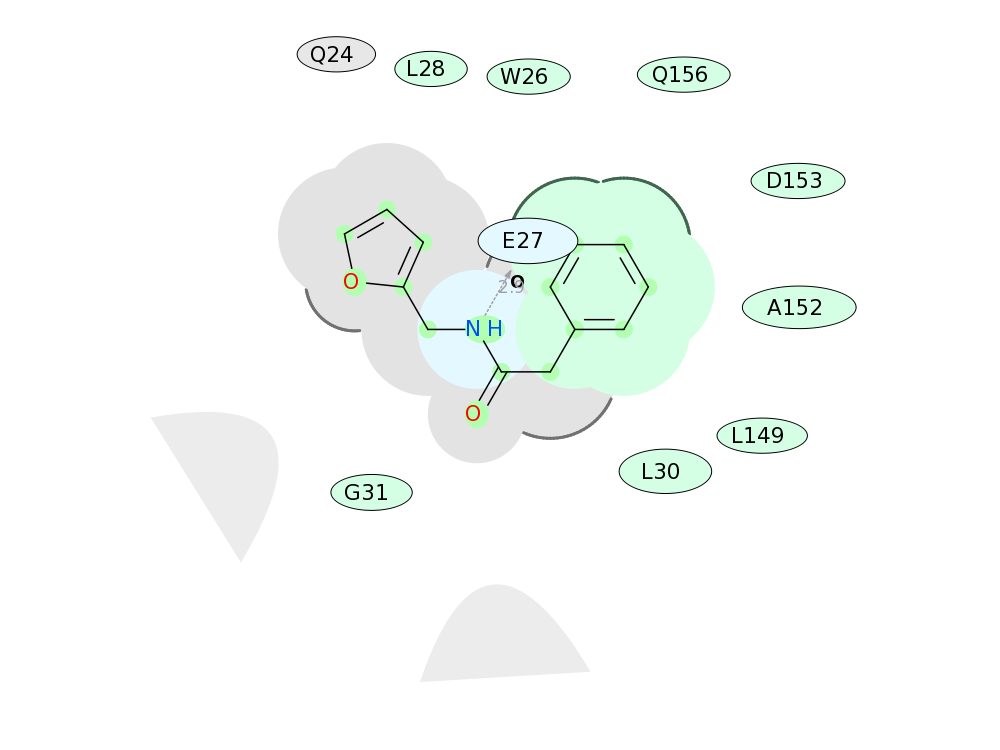


Supplement Figure 5. Furan-amide-benzene (Common structure 4) binds to ApoE4.


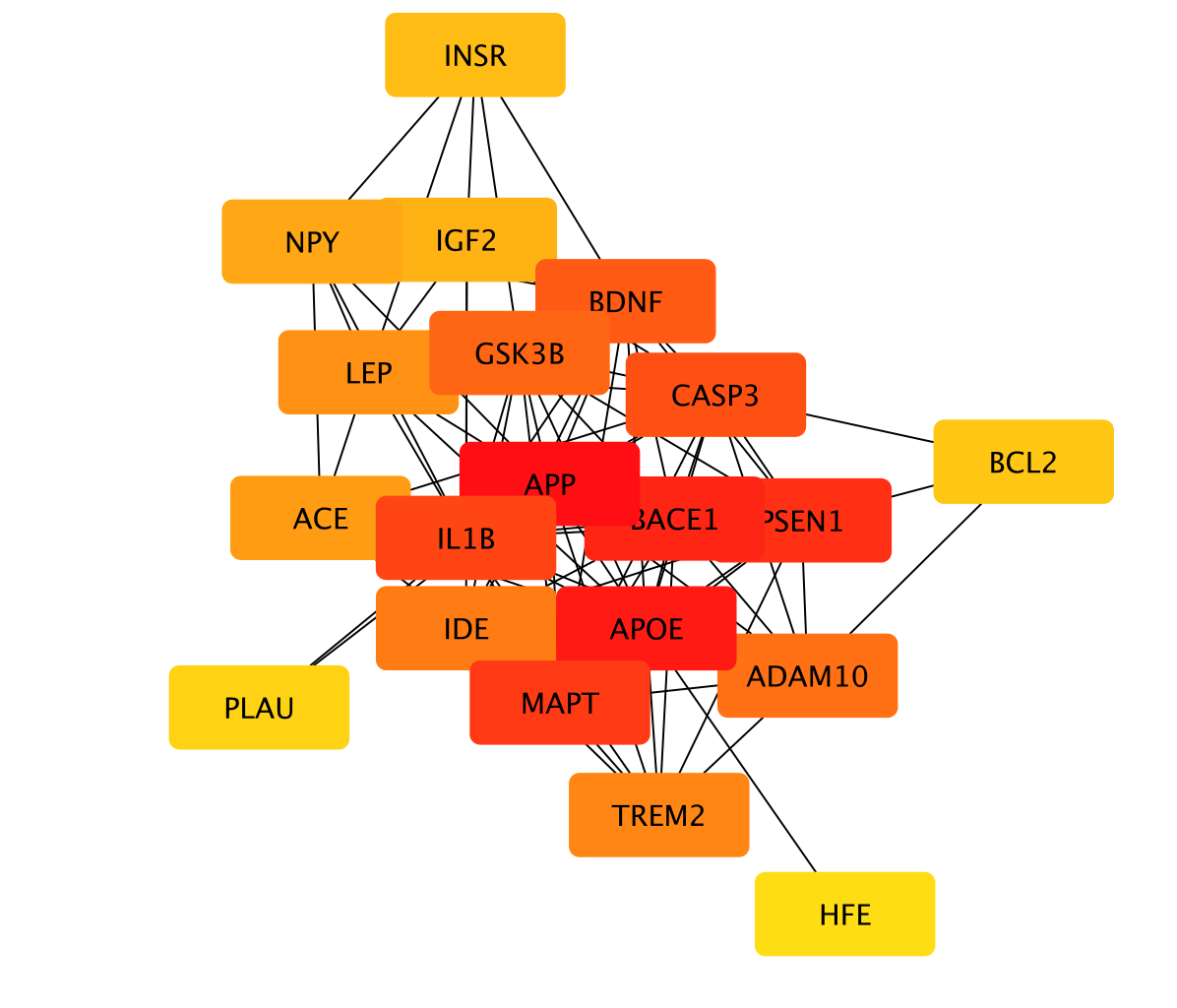


Supplement Figure 6. Network of top 20 Alzheimer’s disease associated genes. the top 20 genes were identified based on the GDA score and the corresponding gene network. This figure includes a brief description of the physiological roles of these genes and their proteins, as well as their ranking based on the CytoHubba analysis. As mentioned, APP is the most important gene, with FDA-approved antibody therapies targeting amyloid-beta. Meanwhile, ApoE ranks as the second most important gene among the well-known AD targets.

Genes list and their physiological role in AD:

1. ACE (angiotensin I converting enzyme) - This enzyme plays a role in blood pressure regulation and the renin-angiotensin system. Due to its effect on cerebral blood flow and amyloid-beta metabolism, it has been implicated in Alzheimer's disease (Kehoe, 2018).
2. APP (amyloid beta precursor protein) - APP is a transmembrane protein that is cleaved to generate amyloid-beta peptides, which aggregate to form amyloid plaques, the defining characteristic of Alzheimer's disease (Selkoe, 2001).
3. ADAM10 (ADAM metallopeptidase domain 10) - This enzyme prevents the formation of amyloid plaques by cleaving APP within the amyloid-beta domain (Kuhn et al., 2010).
4. GSK3B (glycogen synthase kinase 3 beta) is a kinase that participates in multiple signaling pathways, including tau phosphorylation. Important to Alzheimer's disease pathology is abnormal tau phosphorylation (Hernandez et al., 2010).
5. HFE (homeostatic iron regulator) - This protein is involved in iron homeostasis, and mutations in the HFE gene have been linked to Alzheimer's disease due to its potential function in amyloid-beta aggregation (Belaidi & Bush, 2016).
6. APOE (apolipoprotein E) is a lipid transport protein that is essential for cholesterol metabolism. The APOE4 allele contributes to amyloid-beta deposition and is a well-known risk factor for late-onset Alzheimer's disease (Huang & Mucke, 2012).
7. MAPT (microtubule-associated protein tau) encodes the microtubule-stabilizing protein tau. Tau becomes abnormally phosphorylated in Alzheimer's disease, resulting in the formation of neurofibrillary tangles (Iqbal et al., 2010).
8. TREM2 (triggering receptor expressed on myeloid cells 2) is an immune receptor that mediates the activation and phagocytosis of microglia. TREM2 variants are associated with an increased risk of Alzheimer's disease, suggesting that immune dysfunction contributes to disease pathogenesis (Ulland & Colonna, 2018).
9. PSEN1 (presenilin 1) - PSEN1 is part of the gamma-secretase complex, which cleaves APP to produce amyloid-beta peptides. Sherrington et al. (1995) found that mutations in PSEN1 are associated with early-onset familial Alzheimer's disease.
10. PLAU (plasminogen activator, urokinase) is involved in fibrinolysis and remodeling of the extracellular matrix. It is believed to play a role in Alzheimer's disease by affecting amyloid-beta clearance and the integrity of the blood-brain barrier (Tucker et al., 2000).
11. BACE1 (beta-secretase 1) - BACE1 is the beta-secretase enzyme that cleaves APP into amyloid-beta peptides. Due to its function in amyloid-beta synthesis, BACE1 is a significant drug target for Alzheimer's disease (Vassar et al., 1999).
12. IDE (insulin-degrading enzyme) degrades insulin and other substrates, including amyloid-beta. Due to its role in amyloid-beta clearance, IDE dysfunction has been linked to Alzheimer's disease (Kurochkin & Goto, 1994).
13. Interleukin 1 beta (IL1B) - IL1B is a pro-inflammatory cytokine involved in immune responses. Due to its involvement in neuroinflammation and promotion of amyloid-beta production, it has been linked to Alzheimer's disease (Shaftel et al., 2008).
14. The insulin receptor participates in glucose metabolism and insulin signaling. Alzheimer's disease has been linked to impaired insulin signaling, which is commonly referred to as "type 3 diabetes" (de la Monte & Wands, 2008).
15. LEP (leptin) - Leptin is a hormone that regulates the body's energy balance and weight. By modulating amyloid-beta metabolism, it may have neuroprotective effects and play a role in Alzheimer's disease (Greco et al., 2010).
16. NPY (neuropeptide Y) - NPY is a neuropeptide involved in a variety of physiological processes, including regulation of metabolism and stress response. It has been postulated that NPY plays a role in Alzheimer's disease by modulating neuroinflammation and promoting neuronal survival (Ferreira et al., 2015).
17. BCL2 (BCL2 apoptosis regulator) is a crucial anti-apoptotic protein that regulates cell death. Dysregulation of BCL2 expression has been linked to increased neuronal vulnerability and apoptosis in Alzheimer's disease (Czapski et al., 2012).
18. BDNF (brain-derived neurotrophic factor) is a neurotrophin that promotes the survival, differentiation, and plasticity of neurons. Alzheimer's disease has been associated with decreased BDNF levels, and it has been proposed as a potential therapeutic target (Nagahara et al., 2009).
19. CASP3 (caspase 3) - CASP3 is an apoptosis-involved executioner caspase. In Alzheimer's disease, increased CASP3 activation suggests that apoptosis contributes to neuronal loss (Rohn et al., 2001).
20. IGF2 (insulin-like growth factor 2) is necessary for cell proliferation, differentiation, and survival. It has been postulated that it protects against Alzheimer's disease by promoting neuronal survival and decreasing amyloid-beta toxicity (Carro et al., 2002).

Reference list:

1. Kehoe, P. G. (2018). The coming of age of the angiotensin hypothesis in Alzheimer's disease: Progress toward disease prevention and treatment? Journal of Alzheimer's Disease, 62(3), 1443-1466.
2. Selkoe, D. J. (2001). Alzheimer's disease: Genes, proteins, and therapy. Physiological reviews, 81(2), 741-766.
3. Kuhn, P. H., Wang, H., Dislich, B., Colombo, A., Zeitschel, U., Ellwart, J. W., ... & Lichtenthaler, S. F. (2010). ADAM10 is the physiologically relevant, constitutive α-secretase of the amyloid precursor protein in primary neurons. The EMBO journal, 29(17), 3020-3032.
4. Hernandez, F., Langa, E., Cuadros, R., Avila, J., & Villanueva, N. (2010). Regulation of GSK3 isoforms by phosphatases PP1 and PP2A. Molecular and Cellular Biochemistry, 344(1-2), 211-215.
5. Belaidi, A. A., & Bush, A. I. (2016). Iron neurochemistry in Alzheimer's disease and Parkinson's disease: targets for therapeutics. Journal of neurochemistry, 139(Suppl 1), 179-197.
6. Huang, Y., & Mucke, L. (2012). Alzheimer mechanisms and therapeutic strategies. Cell, 148(6), 1204-1222.
7. Iqbal, K., Liu, F., Gong, C. X., & Grundke-Iqbal, I. (2010). Tau in Alzheimer disease and related tauopathies. Current Alzheimer research, 7(8), 656-664.
8. Ulland, T. K., & Colonna, M. (2018). TREM2 — a key player in microglial biology and Alzheimer disease. Nature Reviews Neurology, 14(11), 667-675.
9. Sherrington, R., Rogaev, E. I., Liang, Y., Rogaeva, E. A., Levesque, G., Ikeda, M., ... & George-Hyslop, P. S. (1995). Cloning of a gene bearing missense mutations in early-onset familial Alzheimer's disease. Nature, 375(6534), 754-760.
10. Tucker, H. M., Kihiko, M., Caldwell, J. N., Wright, S., Kawarabayashi, T., Price, D., ... & Estus, S. (2000). The plasmin system is induced by and degrades amyloid-beta aggregates. Journal of Neuroscience, 20(11), 3937-3946.
11. Vassar, R., Bennett, B. D., Babu-Khan, S., Kahn, S., Mendiaz, E. A., Denis, P., ... & Citron, M. (1999). Beta-secretase cleavage of Alzheimer's amyloid precursor protein by the transmembrane aspartic protease BACE. Science, 286(5440), 735-741.
12. Kurochkin, I. V., & Goto, S. (1994). Alzheimer's beta-amyloid peptide specifically interacts with and is degraded by insulin degrading enzyme. FEBS letters, 345(1), 33 -37.
13. Shaftel, S. S., Griffin, W. S., & O'Banion, M. K. (2008). The role of interleukin-1 in neuroinflammation and Alzheimer disease: an evolving perspective. Journal of neuroinflammation, 5(1), 1-12.
14. de la Monte, S. M., & Wands, J. R. (2008). Alzheimer's disease is type 3 diabetes—evidence reviewed. Journal of diabetes science and technology, 2(6), 1101-1113.
15. Greco, S. J., Bryan, K. J., Sarkar, S., Zhu, X., Smith, M. A., Ashford, J. W., ... & Perry, G. (2010). Leptin reduces pathology and improves memory in a transgenic mouse model of Alzheimer's disease. Journal of Alzheimer's Disease, 19(4), 1155-1167.
16. Ferreira, R., Santos, T., Cortes, L., Cochaud, S., Agasse, F., Silva, A. P., ... & Branco, F. (2015). Neuropeptide Y inhibits interleukin-1 beta-induced microglia motility. Journal of neurochemistry, 132(4), 439-452.
17. Czapski, G. A., Cakala, M., Chalimoniuk, M., Gajkowska, B., & Strosznajder, J. B. (2012). Role of nitric oxide in the brain during lipopolysaccharide-evoked systemic inflammation. Journal of neuroscience research, 90(3), 592-602.
18. Nagahara, A. H., Merrill, D. A., Coppola, G., Tsukada, S., Schroeder, B. E., Shaked, G. M., ... & Tuszynski, M. H. (2009). Neuroprotective effects of brain-derived neurotrophic factor in rodent and primate models of Alzheimer's disease. Nature medicine, 15(3), 331-337.
19. Rohn, T. T., Head, E., Nesse, W. H., Cotman, C. W., & Cribbs, D. H. (2001). Activation of caspase-8 in the Alzheimer's disease brain. Neurobiology of disease, 8(6), 1006-1016.
20. Carro, E., Trejo, J. L., Gomez-Isla, T., LeRoith, D., & Torres-Aleman, I. (2002). Serum insulin-like growth factor I regulates brain amyloid-β levels. Nature medicine, 8(12), 1390-1397.
